# Potential Resistance Mechanisms Exhibited by Cystic Fibrosis Patients Against SARS-CoV-2

**DOI:** 10.3390/v17070919

**Published:** 2025-06-27

**Authors:** Yasmin K. Elsharabassi, Nuha T. Swaidan, Mohamed M. Emara

**Affiliations:** Basic Medical Sciences Department, College of Medicine, QU Health, Qatar University, Doha 2713, Qatar; ye1701976@qu.edu.qa (Y.K.E.); ns1201895@qu.edu.qa (N.T.S.)

**Keywords:** SARS-CoV-2, COVID-19, cystic fibrosis, ACE2 receptor, TMPRSS2, CFTR gene, ARDS, resistance factors

## Abstract

Severe acute respiratory syndrome coronavirus 2 (SARS-CoV-2) is the causative agent of the 2019 coronavirus disease pandemic. The virus primarily spreads through person-to-person contact via aerosols and droplets, contributing to high case numbers and related morbidities. SARS-CoV-2 targets the respiratory tract, causing acute respiratory distress syndrome, particularly in immunocompromised individuals such as those with cystic fibrosis (CF). CF is a life-threatening genetic disorder caused by mutations in the CF transmembrane conductance regulator (CFTR) gene, leading to impaired respiratory function and recurrent severe respiratory symptoms. Despite their potential vulnerability, CF patients have shown a lower incidence of severe COVID-19, suggesting protective factors against SARS-CoV-2. Differential expression of the ACE2 receptor, crucial for viral entry, and other host factors, such as TMPRSS2, may play a role in this resistance to SARS-CoV-2. Analyzing the genomics and transcriptomics profiles of CF patients could provide insights into potential resistance mechanisms. The potential resistance mechanisms include blood and extracellular ATP levels, a deleted/dysfunctional CFTR gene, ACE and ACE2 regulation and expression, ACE and ACE2 polymorphism effects, host proteins and SARS-CoV-2 interactions, and SMN1 and ACE/ACE2 interactions. This review discusses the underlying factors and potential resistance mechanisms contributing to CF patients’ responses to SARS-CoV-2 infection. The review provides an opportunity to further investigate future therapy and research through understanding the underlying potential resistance mechanisms exhibited by CF patients against SARS-CoV-2, including ACE and ACE2 polymorphisms.

## 1. Introduction

COVID-19 is a novel disease caused by the severe acute respiratory syndrome coronavirus 2 (SARS-CoV-2), which led to the global outbreak known as the COVID-19 pandemic. The first case was identified in December 2019 in Wuhan, China [1]. Patients with COVID-19 mainly experience respiratory symptoms, such as cough and fever. In severe cases, especially among the elderly and those with underlying conditions like cardiovascular disease, diabetes, chronic pulmonary disorders, or renal disease, the disease can progress to respiratory distress and acute respiratory distress syndrome (ARDS). The pulmonary pathology of COVID-19 is mainly characterized by diffuse alveolar damage and focal reactive hyperplasia of pneumocytes with patchy inflammatory cellular infiltration [2]. SARS-CoV-2 primarily spreads through aerosols and droplets; therefore, understanding its transmission is crucial for assessing the risk of spread and devising effective prevention strategies [3]. Developing effective treatments for COVID-19 is a top priority due to its substantial humanitarian and economic impacts. Researchers globally are employing various technological platforms to develop treatments and vaccines. These technologies include nucleic acid (DNA and RNA) approaches, virus-like particles, peptides, viral vectors (replicating and non-replicating), recombinant proteins, live attenuated viruses, and inactivated viruses [4].

Cystic fibrosis (CF) is a life-threatening disease inherited in an autosomal recessive pattern. It is caused by mutations in the CF transmembrane conductance regulator (CFTR) gene, which encodes an ion channel protein that affects multiple systems in the human body [5]. According to a review published in 2019, the total number of CF patients in Qatar is 82, comprising 34 adults and 48 children [6]. In Qatar, the *CFTR* I1234V mutation is the most common mutation among CF patients [7]. CF leads to the secretion of abnormally thick mucus and produces defects in electrolyte transport [8]. Patients with CF often suffer from recurrent lung infections at a young age caused by pathogens such as *Staphylococcus aureus*, *Haemophilus influenzae*, and the mucoid phenotype of *Pseudomonas aeruginosa*. Several tests are performed to evaluate CF patients, including the pulmonary function test (PFT), which is the gold standard for assessing disease progression, and computed tomography (CT) scoring systems to quantify structural abnormalities [7]. The primary diagnostic test for CF is the sweat chloride test [5]. A sweat chloride value of ≥60 mmol/L confirms a CF diagnosis, while a value of ≤29 mmol/L makes a CF diagnosis unlikely [5]. Values between 30 and 59 mmol/L necessitate further testing to confirm the diagnosis [5].

According to data, a small number of CF patients in countries such as France, the United Kingdom, and Germany have been reported to have SARS-CoV-2 infection without it affecting the severity of CF disease [9]. Multiple studies indicate that the incidence of SARS-CoV-2 among the CF population is lower than in the general population. For instance, a study conducted in Belgium assessed the anti-SARS-CoV-2 IgM and IgG levels of 149 CF patients between April and May 2020 [10]. The results showed a lower seroprevalence in CF patients (2.7%) compared to the general population (4.9%), suggesting a lower infection rate. Although nearly 60% of severe acute pulmonary disorders in CF patients are attributed to viral infections, preliminary reports suggest that SARS-CoV-2 infection does not worsen their medical condition [10]. This information highlights the need for further investigation into the potential factors that may protect CF patients from acquiring SARS-CoV-2 and result in less severe infections among those who contract the virus.

Previous studies have demonstrated that CF patients are more susceptible to respiratory viruses due to weakened immune responses, leading to severe infections and increased morbidity [11]. During the 2019 Influenza A (H1N1) pandemic, CF patients had high morbidity and mortality [11]. A study showed that H1N1 infection caused significant morbidity to CF patients, including two-thirds of the patients requiring intravenous antibiotic therapy, half of them requiring hospitalization, and 30% needing supplemental oxygen treatment during the period of infection [12]. Respiratory syncytial virus infections are common in both adult and pediatric CF patients and can cause severe symptoms. RSV infection can result in both upper and lower respiratory disease, and it is aggressive in young infants with CF, which can lead to high respiratory morbidity [13]. However, some studies suggest that CF patients have more favorable outcomes with COVID-19 compared to other respiratory infections. This has led to the hypothesis that certain protective factors in CF airway cells may help prevent or reduce the severity of SARS-CoV-2 infections [11]. This review will explore these potential protective factors.

## 2. Cystic Fibrosis Genetics

**Figure 1:**
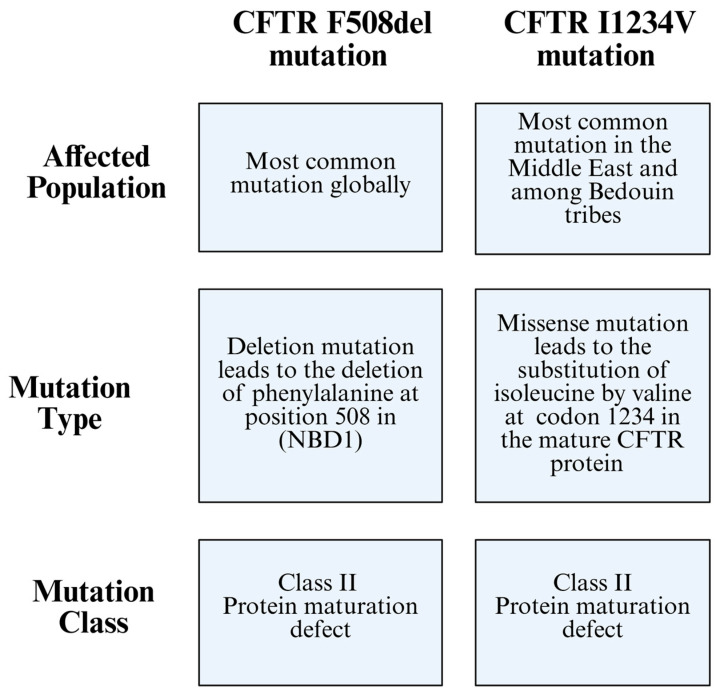
Comparison between the CFTR F508del mutation and the CFTR I1234V mutation, highlighting differences in the affected population, mutation type, and mutation class.

CF is a monogenic disorder affecting the CFTR gene, which encodes the CFTR protein. The F508del mutation is the most frequent CF-causing mutation worldwide; however, its prevalence varies among populations (Figure 1) [14].

CF affects multiple organs, including the airways of the lung, pancreas, sweat glands, intestines, and male reproductive tract. CFTR functions as a chloride channel regulated by cyclic adenosine monophosphate (cAMP)-dependent phosphorylation, and mutations in CFTR lead to ion transport disturbances in CF patients. The CFTR genotype is associated with the severity of pancreatic exocrine disease and, to a lesser extent, with sweat chloride concentration. However, no clear relationship exists between lung function and CFTR genotype [15].

**Figure 2:**
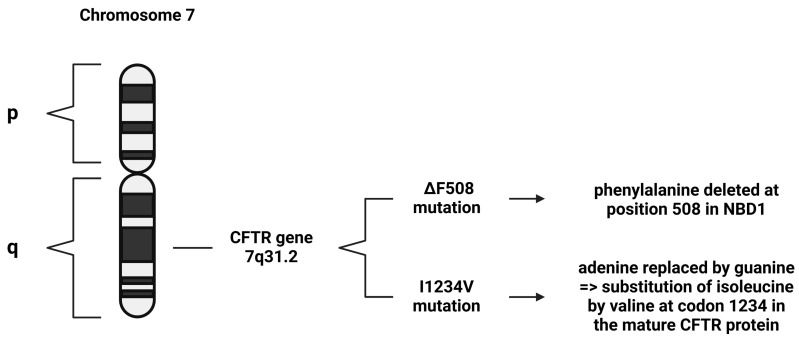
Demonstration of different CFTR mutations. The ΔF508 mutation is the most common CF mutation worldwide, and the I1234V mutation is the most common CF mutation in Qatar.

Several mutations have been found within the CFTR gene, and these are the main cause of the disease. These CFTR variants include L88X, M152V, F508del, G551D, E831X, W1282X, and E1418X. The ΔF508 mutation is the most common mutation in CF, characterized by the deletion of phenylalanine at position 508 in nucleotide-binding domain 1 (NBD1) (Figure 2). This mutation impairs the protein folding, plasma membrane expression, function, and stability of the CFTR protein [14]. Along with this mutation, the L88X and M152V variants produce immature, dysfunctional proteins that undergo degradation via the ubiquitin–proteasome system [16]. In contrast, the G551D variant produces a mature but dysfunctional protein. Similarly, the E831X and E1418X variants produce mature proteins with reduced functionality. On the other hand, the W1282X variant results in unstable mRNA, leading to the absence of protein production [16].

The I1234V mutation is a missense mutation, where adenine is replaced by guanine, which results in the substitution of isoleucine by valine at codon 1234 in the mature CFTR protein (Figure 2) [17]. According to a World Health Organization (WHO) report, the I1234V mutation is rarely encountered globally; however, it is frequently encountered in the Middle East, specifically among Bedouin tribes [6].

CF mutations are classified, based on their cellular phenotype, into six classes. Class I is characterized by a protein synthesis defect, Class II is characterized by a maturation defect, Class III is characterized by a gating defect, Class IV is characterized by a conductance defect, Class V is when protein quantity is reduced, and Class VI is when protein stability is reduced [18]. A ΔF508-CFTR mutation is classified as a Class II mutation, where it damages CFTR conformational maturation, leading to its targeting for premature ER-associated degradation [18]. A previous study proved that I1234V mutation can be characterized as a Class II mutation after visualizing its misfolded CFTR protein structure (Figure 1) [19].

A global study found 105,352 CF diagnoses across 94 countries, with 47,650 cases in Europe, 37,002 in North America, 3652 in Australasia, 5349 in Asia, and 1665 in Africa [20]. CF is often diagnosed through screening programs and genetic analysis, which can identify homozygous patients with mild or no symptoms [20]. In Qatar, many pediatric CF patients have the CFTR I1234V mutation, showing mild to moderate symptoms, while non-Qatari patients of non-Arab Asian descent tend to have more severe respiratory issues and earlier Pseudomonas aeruginosa colonization [11].

Genetic factors significantly influence the establishment of chronic *Pseudomonas aeruginosa* infection and the age of onset in CF patients. Transforming growth factor beta 1 (TGFB1) is involved in tissue repair and is a modifier of CF lung disease, with variants linked to asthma and chronic obstructive pulmonary disease (COPD); both conditions exhibit symptoms similar to CF [15].

## 3. Respiratory Viral Infections Associated with Cystic Fibrosis

CF patients frequently experience recurrent respiratory infections that damage the lungs and lead to progressive deterioration of lung function, often resulting in early mortality [21]. Various respiratory viruses contribute to serious clinical symptoms, and these respiratory viruses cause substantial morbidity, particularly in infants, the elderly, and immunocompromised individuals [21]. The upper respiratory tract is typically colonized by a variety of microorganisms known as the normal flora, while the lower respiratory tract remains sterile under healthy conditions. A breakdown in any of the innate defense mechanisms can lead to infections in the lower respiratory tract [22]. Viral infections in CF patients are particularly common during early childhood [23]. A previous study performed on CF infants exploring the impact of viruses on lung disease demonstrated that respiratory viruses were a primary cause of hospitalization during the first year of life [23]. Viral respiratory infections in CF infants often lead to more severe and prolonged lower respiratory tract symptoms [24]. In the coming sections, different respiratory viruses associated with cystic fibrosis will be discussed, with SARS-CoV-2 being discussed in detail.

### 3.1. Human Rhinovirus and Other Predominant Respiratory Viruses

**Figure 3:**
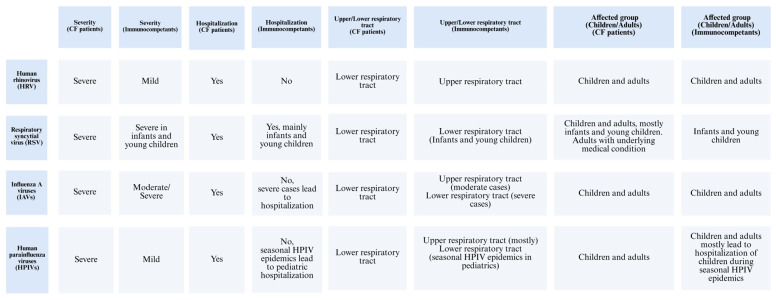
Comparison of different respiratory viruses and their impact on CF patients versus immunocompetent individuals.

In CF patients, viral infections are predominantly caused by human rhinovirus (HRV), parainfluenza viruses, coronavirus, respiratory syncytial virus (RSV), human metapneumovirus (HMPV), influenza viruses, and adenovirus [24]. Among these, RSV, HRV, and the influenza virus are the most common causes of viral respiratory infections [25]. These viruses tend to result in more severe clinical outcomes in individuals with chronic airway diseases [26]. Pulmonary exacerbations in CF patients are commonly triggered by lower respiratory viral infections [27]. Among these, HRV is recognized as the most frequently detected virus in the airways of both adults and children with CF [27]. Infection with HRV in CF patients is associated with significant clinical impacts, such as increased hospitalization rates and greater reliance on intravenous antibiotic treatment [27]. HRV was identified as the most frequent virus in adults with CF, where it is typically the main cause of the common cold but is also associated with acute asthma attacks in patients with airway diseases (Figure 3) [26].

### 3.2. Respiratory Syncytial Virus

RSV is a well-known cause of serious respiratory tract infections in infants and young children [28]. Additionally, it can lead to significant clinical impact on adults with cardiopulmonary diseases, the fragile elderly, and those who are severely immunocompromised [28]. RSV, like other viral respiratory infections, is linked to acute pulmonary exacerbations in CF patients [29]. Data show that CF infants who acquire RSV lower respiratory tract infection often require hospitalization for acute respiratory illness, accounting for roughly one-third of such admissions [29]. Although viral infections are not highly prevalent in CF patients, their clinical impact is often more severe, leading to acute respiratory complications [29]. RSV is the most commonly reported virus during respiratory exacerbations in young children, with incidence rates ranging from 9% to 58% (Figure 3) [29].

### 3.3. Influenza Viruses

Influenza viruses are a major cause of respiratory illness in humans, leading to significant public health concerns [30]. Among the influenza viruses, the Influenza A virus (IAV) is responsible for recurring epidemics each year [30]. While influenza viruses often cause moderate respiratory illness, they can also result in more severe infections, including lower respiratory tract involvement, which may progress to pneumonia, ARDS, and, in severe cases, respiratory failure and death [30]. On average, seasonal IAV infections affect up to 20% of children and 10% of adults annually [30]. The 2009 H1N1 pdm09 pandemic had a pronounced impact on younger age groups, particularly those with chronic respiratory conditions such as CF [31]. During this time, 64% of the reported deaths occurred in children with severe pre-existing disorders, including respiratory diseases [31]. Furthermore, a population-based study found a significant association between regional influenza activity and an elevated risk of pulmonary exacerbations in both children and adults with CF [32]. While influenza pneumonia can directly cause CF lung exacerbations, this is relatively rare in the general population (Figure 3) [32].

### 3.4. Human Parainfluenza Viruses

Human parainfluenza viruses (HPIVs) are key pathogens that cause respiratory diseases in both children and adults, presenting clinical symptoms such as colds, croup, bronchiolitis, and pneumonia [33]. Seasonal epidemics of HPIVs are responsible for 40% of pediatric hospitalizations related to lower respiratory tract infections (LRTIs) and 75% of croup cases [33]. HPIV infection typically begins in the nose and oropharynx, then spreads to the lower airways [33]. In immunocompetent individuals, HPIV infections are generally mild and transient. Although there are no reports about this virus infection in CF patients, those with asthma and COPD have experienced severe exacerbations of respiratory symptoms due to HPIV infection [34]. Among children under five years, HPIVs are reported as the second most common cause of acute respiratory tract infections, following RSV, and are responsible for approximately 17% of hospitalizations (Figure 3) [34].

### 3.5. Severe Acute Respiratory Syndrome Coronavirus 2

SARS-CoV-2 is a respiratory virus associated with cystic fibrosis patients, as they exhibit respiratory symptoms that can be severe when the viral infection is acquired [35]. As mentioned previously, the first COVID-19 case was reported in Wuhan, China, at the end of 2019 [36]. While COVID-19 cases continue to be reported globally, seroprevalence surveys in the United States suggest that, even after adjusting for potential false positives and negatives, the rate of previous exposure to SARS-CoV-2, confirmed by seropositivity is 6 to 24 times higher than the incidence of reported cases [37]. SARS-CoV-2 primarily spreads through respiratory transmission from person to person. The initial stage of SARS-CoV-2 infection is the most contagious, characterized by the highest levels of viral RNA present in the upper respiratory tract [38]. The immune response to SARS-CoV-2 infection includes both cellular and humoral immunity. Studies indicate that cell-mediated immunity plays a role in SARS-CoV-2 infection, with SARS-CoV-2-specific helper T cell (CD4) and cytotoxic T cell (CD8) responses observed in individuals who have recovered from COVID-19 and those who have been vaccinated [39]. Humoral immunity is characterized by serum antibodies that possess neutralizing activity through targeting the receptor-binding domain of the viral spike protein; therefore, these antibodies play a crucial role during SARS-CoV-2 infection, and they can be detected after the infection [40]. The SARS-CoV-2 genome sequence is similar to that of other coronaviruses, consisting of approximately 29,903 nucleotides of linear single-stranded RNA (ssRNA) containing 14 open reading frames (Orfs). The structural proteins of SARS-CoV-2 are encoded by the S, E, M, and N genes, while accessory proteins are encoded by the Orf3a, Orf3b, Orf6, Orf7a, Orf7b, Orf8, Orf9b, Orf9c, and Orf10 genes [41]. According to studies, mutations in these structural proteins contribute to the evolution of SARS-CoV-2 variants. Since 2023, the Centers for Disease Control and Prevention (CDC) recognizes only lineages of the Omicron variant as variants of concern (VOCs) [42]. The Omicron variant, initially identified as the BA.1 sub-lineage, harbors 37 mutations in the S protein and was rapidly classified as a VOC due to its enhanced transmissibility and immune escape potential [43]. Since then, multiple Omicron subvariants, including BA.2, BA.5, and XBB, have emerged, each carrying distinct sets and numbers of spike mutations. These sub-lineages continue to be monitored for their epidemiological relevance and potential impact on vaccine effectiveness [44,45]. Upon viral entry into the host cell, viral RNA is translated to produce viral replicase polyproteins, which then self-cleave to form non-structural proteins. These non-structural proteins form a replicase/transcriptase complex that facilitates the transcription and replication of genomic and sub-genomic mRNA. Structural proteins, including S, E, M, and N, are produced from sub-genomic mRNA. After assembly of genomic RNA and structural proteins in the ER–Golgi apparatus, newly formed SARS-CoV-2 viral particles are released from the cell via an exocytosis mechanism [45]. The processes of replication and transcription of SARS-CoV-2 genomic RNA are carried out by nsp12, which has RNA-dependent RNA polymerase (RdRp) activity [41].

A study examined the differential responses of bronchial epithelial cells from various donors, with and without CF, to viral infections, focusing on SARS-CoV-2. The study results revealed that CF epithelial cells demonstrated lower levels of SARS-CoV-2 replication for both the D614G and Omicron BA.1 variants compared with cells from individuals without CF, which is a pattern not detected with IAV [46]. The CF epithelial cells used in the study are from patients with the F508del mutation or nonsense mutations. The F508del-CFTR protein is misfolded and degraded, leading to the initiation of an unfolded protein response. However, CF epithelial cells from patients with nonsense mutations also demonstrated reduced viral infection levels, indicating factors other than the endoplasmic reticulum stress and proteasome-dependent degradation pathways linked to the F508del mutation might contribute to the reduced susceptibility to SARS-CoV-2 infection in CF patients [46].

## 4. SARS-CoV-2 Invasion Mechanisms and Pathogenicity

Coronavirus is an enveloped and non-segmented virus with a positive-sense single-stranded RNA genome belonging to the Coronaviridae family. The virus has multiple spikes on its surface, with the spike (S) glycoprotein playing a vital role in viral entry into the host cell by attaching to cellular receptors expressed by the host cell [47]. Another component of the coronavirus is the virion, which consists of genomic RNA and a protein capsid encapsulated within a nucleocapsid. This nucleocapsid is surrounded by a phospholipid bilayer, which is primarily composed of the spike glycoprotein trimmer (S) and hemagglutinin-esterase (HE) [47].

There are four genera of coronaviruses (CoV): α-, β-, γ-, and δ-CoV. Both SARS-CoV and Middle East respiratory syndrome coronavirus (MERS-CoV) belong to β-CoVs and can cause severe and often fatal respiratory tract infections [47]. Experimental results indicate that both SARS-CoV and SARS-CoV-2 infect humans by utilizing the angiotensin-converting enzyme 2 (ACE2) receptor [47]. SARS-CoV-2 infection progresses through three phases: an asymptomatic phase, a non-severe symptomatic phase, and a severe, potentially lethal phase characterized by hypoxia. ARDS is the primary cause of death in COVID-19, resulting from a cytokine storm, which is an uncontrolled systematic inflammatory response. This storm involves the release of pro-inflammatory cytokines and chemokines by various immune cells [48]. Coronaviruses enter the host cells through utilization of the clathrin-mediated endocytosis (CME) mechanism, which occurs when the virion binds to host receptors, such as ACE2, and host proteases, like transmembrane serine protease 2 (TMPRSS2) or furin [49]. Recent studies suggest that SARS-CoV-2 is present in the central nervous system (CNS) of COVID-19 patients. At the same time, it has been proven that neurons use a CME mechanism similar to non-neuronal cells, with endocytosis occurring at synapses involving neuron-specific isoforms of CME endocytic proteins [49]. The N-linked glycosylated trimeric spike protein uses N-terminal signals to enter the host endoplasmic reticulum. The S protein consists of two subunits: S1, which acts as the receptor binding domain, and S2, which provides structural support by forming the stalk necessary for the spike’s structure [47].

Most viruses utilize the host cell’s endocytic mechanisms to enter the host cell. The primary goal of virus entry is to transfer its genome into the host cell to enable replication. The virus entry process begins with attachment, where the virus particle binds to the cell surface. Next is penetration, where the virus introduces its genome and accessory proteins into the cell cytosol. Following this, uncoating and replication occur, during which the viral genome is released from the capsid, allowing viral RNA or DNA to be transcribed and replicated within the host cell [50]. Then, assembly comes as the next step, where viral proteins are assembled. Finally, the new viral particles are released from the host cell [51]. The mechanisms of SARS-CoV-2 neuro-invasion are not fully understood, but its spread in the CNS can be considered in light of the known routes of SARS-CoV-2 transmission. Potential mechanisms include hematologic spread, retrograde transport from the peripheral nervous system (PNS), and blood–brain barrier (BBB)-mediated spread [49]. SARS-CoV-2 may enter the CNS by directly infecting olfactory peripheral nerve terminals and other cranial nerves. Additionally, an interaction between ACE2 and integrin β-1 may play a crucial role in the entry and spread of CoV-2 in the CNS, as integrin β-1 is vital for neural functions such as myelination, axon guidance, and cell adhesion [49].

## 5. Potential Resistance Factors Against SARS-CoV-2 in CF Patients

**Figure 4:**
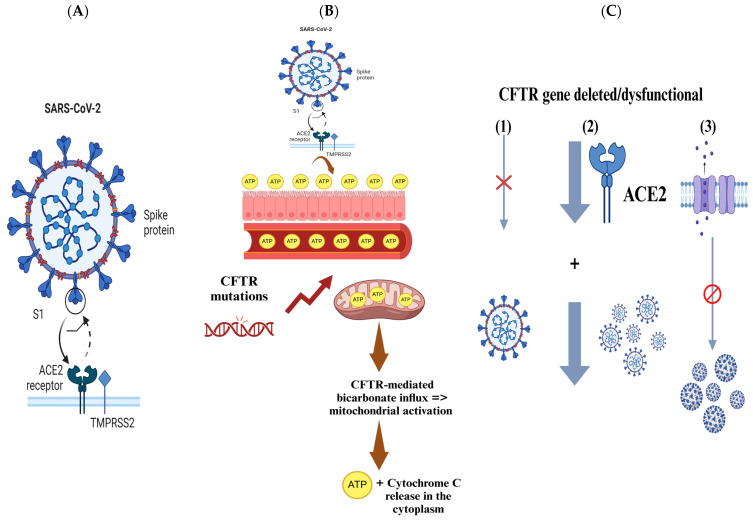
The figure presents key insights into the interactions between SARS-CoV-2 and CF patients, focusing on genetic factors and mechanisms that may influence viral infection and outcomes. (**A**) SARS-CoV-2 enters host cells via the S glycoprotein binding to the ACE2 receptor, with proteases like TMPRSS2 and furin aiding viral membrane fusion. (**B**) Elevated ATP levels in CF patients might provide protection against SARS-CoV-2. ATP, which plays a crucial role in metabolism and signaling, is produced in greater quantities in CF patients due to CFTR gene mutations. This increased ATP could help combat viral infections by supporting cellular function. (**C**) CFTR gene mutations or deletions can reduce SARS-CoV-2 viral load by inhibiting replication. Additionally, CFTR dysfunction may alter ACE2 abundance and glycosylation, decreasing infection efficiency and disrupting the ionic regulation, further limiting viral replication.

As mentioned previously, the S glycoprotein of SARS-CoV-2 facilitates viral entry into host cells by binding to the ACE2 receptor, which is abundantly expressed in pulmonary epithelial cells and intestinal enterocytes [52]. Following this binding, the S protein undergoes processing by several proteases, including TMPRSS2 and furin, which are essential for priming the S protein and facilitating the fusion of viral and host cell membranes [52]. CF is characterized by severe lung function impairment, making it a high-risk comorbidity for COVID-19 due to the known impact of other viral infections, such as RSV and H1N1, which can rapidly deteriorate lung function and increase mortality in CF patients [52]. Surprisingly, several studies from CF cohorts in Belgium, France, Spain, Germany, and Italy have reported that these patients tend to experience mild symptoms upon SARS-CoV-2 infection [52]. Therefore, it is hypothesized by these reports that specific host factors associated with CF may affect susceptibility to SARS-CoV-2 (Figure 4A) [52].

### 5.1. Adenosine Triphosphate (ATP)

Previous experimental studies using ischemia/reperfusion models showed that adenosine and its agonists have a role in blocking infiltration, trafficking, activation of PMNs, and production of superoxides, with reperfusion damage reduction. For this reason, adenosine could be used to treat respiratory medical conditions, such as acute lung injury (ALI) and ARDS [53]. Adenosine promotes cellular response to hypoxia in the case of lung inflammation. Moreover, it promotes the reduction of extravasation of proteins and cytokines in the alveolus with decreased unfiltered neutrophils [53]. In the Great Metropolitan Bianchi Melacrino Morelli (GOM) Hospital in Reggio Calabria, Italy, inhaled adenosine was used as a treatment in a clinical case of a hospitalized COVID-19 patient. CT scans of the patient after treatment showed significant improvement compared to CT scans before treatment [53].

A study demonstrated that virus-infected cells can release ATP at a high level as a “danger signal”. Subsequently, by facilitating IFN-β expression in P38/JNK/ATF-2 signaling pathways, extracellular ATP protects the host against DNA and RNA virus infections [54]. Part of the study was performed using vesicular stomatitis virus (VSV)-infected RAW 264.7 and L929 cells to detect ATP release and levels (ATP-release assay). The study results proved that extracellular ATP is significantly increased in viral infection. Additionally, ATP release is a frequent phenomenon in virus infection in vitro and in vivo [54]. MTS assay for cell viability was performed using RAW 264.7 cells and 293T cells, and the result showed that cell viability of VSV-infected RAW 264.7 and 293T was enhanced by ATP, which indicates that extracellular ATP plays an essential role in protecting virus-infected cells, while uninfected RAW 264.7 and 293T cells demonstrated a slight change in cell viability at low concentrations. Therefore, ATP protection in viral infection is independent of cell proliferation [54].

CF patients exhibit elevated levels of blood and extracellular ATP, which may explain why certain solid tumors, such as malignant melanoma that express purine receptors, are rarely seen in CF patients [55]. In addition, CF appears to provide enhanced protection against SARS-CoV-2 infection [55]. Patients with CF who are heterozygous carriers of CFTR gene mutations show clinical benefits, including improved infection survival. There is evidence that suggests that the CF carrier state may provide protection against diseases like typhoid, cholera, and tuberculosis [55]. Recent studies have also indicated that individuals with homozygous CFTR mutations may have a survival advantage against SARS-CoV-2 [55]. This is thought to be linked to ATP’s essential role in both intracellular metabolism and extracellular signaling, which may help explain the enhanced survival outcomes in CF patients with SARS-CoV-2 infection (Figure 4B) [55].

Several facts support the hypothesis suggested above. One of the key facts is that CFTR gene mutations enhance mitochondrial ATP production [55]. Studies conducted by Ballard and her team using myocytes confirmed that CFTR modulates ATP release via pannexin-1 channels that are present within the plasma membrane [55]. These studies demonstrated that mild acidosis increases CFTR-mediated bicarbonate influx, triggering mitochondrial activation, which in turn leads to the release of ATP and cytochrome C into the cytoplasm [55]. This process results in the opening of plasma membrane pannexin-1 channels, allowing ATP to be released to the extracellular space, establishing a connection between CFTR presence in the plasma membrane, mitochondrial ATP production, and ATP release (Figure 4B) [55].

ATP depletion could also occur through mechanisms related to COVID-19 [55]. SARS-CoV-2 infection has been associated with blood clotting in organs such as the lungs and brain [55]. Micro-clots in the lung capillaries may lower the arterial oxygen pressure (PaO_2_), which in turn reduces local hemoglobin oxygenation and ATP production in local tissues [55].

In general, viral infections, including SARS-CoV-2, may deplete ATP levels [55]. When systemic ATP levels drop below a critical threshold, the immune response is impaired, as ATP is required for both energy distribution and immune system function [55]. In case of sufficient ATP, an efficient immune response typically develops within 4 to 6 weeks post-infection with SARS-CoV-2 [55]. However, when ATP is depleted, the immune response may be weakened [55]. Therefore, the elevated ATP levels in CF patients may provide them with a protective advantage in the context of COVID-19 infections (Figure 4B) [55].

### 5.2. Deleted/Dysfunctional CFTR Gene

A previous study compared SARS-CoV-2 infection in wild-type (wt/wt) human bronchial epithelial cells and those with CFTR gene modifications [56]. The results revealed that SARS-CoV-2 infection is significantly inhibited in cells where the CFTR gene is either deleted or dysfunctional [56]. Furthermore, human bronchial epithelial cell lines with targeted CFTR gene deletion exhibited a stronger basal downregulation of ACE-2 expression compared to the CFBE41o-∆F cell line, which only showed a mild downregulation [56]. The study also confirmed that the decrease in viral replication in cells with CFTR mutation or deletion is related to the loss of CFTR function. This was supported by treatment with the CFTR inhibitor (IOWH-032), which impaired the viral replication cycle, and by VX-661+VX-445 treatment, which improved SARS-CoV-2 viral replication in CFTR-modified cells by partially restoring CFTR functionality (Figure 4C) [56].

In vitro analysis revealed that alteration or complete deletion of the CFTR gene significantly reduced the SARS-CoV-2 viral load in both cellular supernatant and within the cells [56]. The data suggest that the severity of SARS-CoV-2 infection may depend on the complete loss of CFTR expression or activity, as the highest inhibition of viral replication occurred when CFTR was entirely deleted [56]. Several studies have highlighted several protective factors against SARS-CoV-2 infection in people living with CF [56]. Mutations in the CFTR gene may alter the abundance or the glycosylation pattern of ACE-2 and TMPRSS-2 proteins, which could reduce the effects of SARS-CoV-2 infection by changing the pH of organelles in the protein secretory pathway (Figure 4C) [56].

Interestingly, the study found that ACE-2 expression was slightly lower in ∆F cells than in WT CFBE41o cells, indicating a potential role for ACE-2 in limiting SARS-CoV-2 entry into host cells [56]. In CFTR-inhibited CFBE41o-WT cells, ACE-2 expression appeared to be upregulated over time following SARS-CoV-2 infection; however, the viral load was lower compared to CFBE41o-∆F cells [56]. These findings suggest that the disruption of CFTR function and the resulting ionic imbalance may play a crucial role in affecting viral replication beyond the expression of the classical SARS-CoV-2 ACE-2 receptor (Figure 4C) [56].

Disruption of ionic regulation due to CFTR dysfunction may interfere with the viral replication cycle and cause intracellular pH changes that alter protein assembly and structure [56]. Multiple studies have pointed to CFTR’s role in endosome trafficking and fusion, which is the primary entry route for many enveloped viruses, including SARS-CoV-2 [56]. Overall, the data indicate that the SARS-CoV-2 infection rate is lower in CFTR-defective cells compared to normal cells [56]. Although several mechanisms might contribute to the inhibition of viral replication in CFTR-defective cells, ACE-2 regulation is one of these mechanisms [56]. Additionally, findings from CFTR ablation and inhibitor treatments suggest that ionic dysregulation due to CFTR loss may be an essential protective mechanism against SARS-CoV-2 infections, providing potential new pharmacological insights for SARS-CoV-2 treatment (Figure 4C) [56].

A study result in agreement with a multinational report across eight countries enrolling 40 CF patients who were positive for SARS-CoV-2 revealed that the baseline lung inflammatory status may have modulated CF patients’ susceptibility to SARS-CoV-2 [57]. The observation of the chronic presence of neutrophils in CF lungs is noteworthy because it may play an essential role in the control of infections and tissue repair. Notably, neutrophils have the ability to counteract infections via phagocytosis and/or the release of neutrophil extracellular traps, thus contributing to modulating CF patients’ susceptibility to SARS-CoV-2 [57].

According to previously reported cases in medical literature, CF patients contract SARS-CoV-2 infection at lower rates compared to the general population. The physiological factors that may mitigate COVID-19 in CF patients could be the localized respiratory tract reduction of IL-6, thick secretions in the respiratory tract, existing microbiota, and elevated autophagy induction [58]. Moreover, therapeutic/interventional factors, such as neutrophil elastase inhibitors and azithromycin, may mitigate COVID-19 in CF patients. The localized constitutive reduction of IL-6 in CF patients’ respiratory tracts could be a protective factor against SARS-CoV-2 infection-related cytokine storms. CFTR protein cleavage may be involved in the SARS-CoV-2 molecular pathology, even though the subsequent effect on disease outcomes in CF patients is unclear [58].

### 5.3. ACE and ACE2 Regulation and Expression

As indicated earlier, the ACE2 receptor is a key facilitator for SARS-CoV-2 entry into host cells, which occurs through membrane fusion and endocytosis [59]. Individuals with active COVID-19 exhibit elevated levels of circulating ACE2, which remain high even after recovery [59]. Notably, patients with risk factors for severe COVID-19, such as chronic conditions, expressed higher levels of circulating ACE2 [59]. The extensive distribution of ACE2 across various tissues may facilitate viral invasion and increase the risk of damage to susceptible organs [60]. In contrast, ACE2 plays a protective role in non-communicable diseases like autoimmune, cardiovascular, metabolic, and gastrointestinal disorders, where it helps counteract inflammation and disease progression [60]. Thus, ACE2 has a dual role—physiologically protective, yet virally conducive [60].

Interestingly, some studies have reported unexpectedly mild COVID-19 symptoms in CF patients [11]. Research suggests that CF airway epithelial cells demonstrate altered intracellular processes during host defense against SARS-CoV-2, which may serve as protective factors against potential viral infection and replication [11]. Previous studies have shown that CF patients have an impaired innate immune response to respiratory viruses [11]. While elevated interleukin-6 (IL-6) levels are linked with severe COVID-19 and higher mortality, CF patients show lower IL-6 levels in their airway epithelia, which might offer protection against severe SARS-CoV-2 infection [11].

A previous study confirmed that SARS-CoV-2 spike protein downregulates ACE2 expression, therefore leading to cardiovascular stress that results in lung injury [61]. Nevertheless, in CF patients, ACE polymorphisms and the downregulation of ACE2 expression may contribute to the variability in COVID-19 severity [11]. A study involving 180 CF patients examined the effect of biallelic ACE polymorphisms, revealing that patients with biallelic ACE deletions experienced earlier onset of clinical symptoms and were at higher risk of lung deterioration compared to patients with monoallelic or biallelic insertions [11]. According to this study, ACE2 expression was significantly reduced after viral infection [61]. This result was confirmed by analyzing 26 viral proteins from SARS-CoV-2 to detect the proteins that were responsible for the reduction in ACE2. These viral proteins were transfected in hACE2-HeLa cells, and the ACE2 protein level was detected by WB [61]. A remarkable ACE2 reduction was detected in spike-expressed hACE2-HeLa cells compared to other viral proteins [61]. Additionally, another protective factor in CF patients could be the thick mucus secretions and the pre-existing microbiota in their airways, which could impede viral entry [11].

According to a recent study, it has been confirmed in cell cultures that soluble ACE2 fused to Ig, or a nonspecific protease inhibitor called camostat mesylate, can inhibit pseudovirus infections bearing the S protein of SARS-CoV-2. High doses (100 µg/mL) of camostat mesylate partially reduced SARS-CoV-2 growth, which is expected based on previous studies using different viruses [62]. To prove that clinical-grade hrsACE2 can interfere with SARS-CoV-2 infections, an experiment was performed by infecting Vero-E6 cells (cells used for SARS-CoV-2 isolation) with different numbers of SARS-CoV-2: 10^3^ plaque-forming units (PFUs; MOI 0.02), 10^5^ PFUs (MOI 2), and 10^6^ PFUs (MOI 20) [62]. Afterwards, viral RNA was purified from cells and assayed by qRT-PCR, considering it as a marker for replication. Infection of cells in the presence of hrsACE2 for 1 h, followed by washing and incubation without hrsACE2, significantly inhibited SARS-CoV-2 infection of Vero-E6 15 h post-infection [62].

## 6. SARS-CoV-2 Resistance Mechanisms in CF Patients

### 6.1. ACE and ACE2 Polymorphism Effects

**Figure 5:**
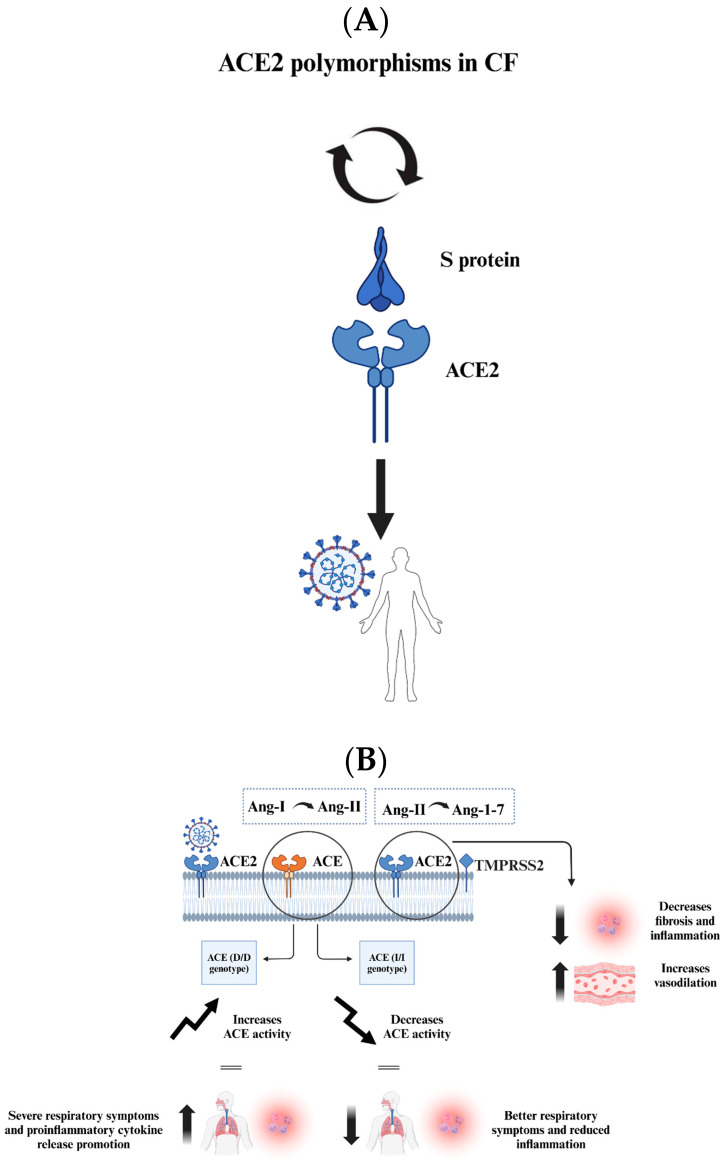
The figure presents key insights into the interactions between SARS-CoV-2 and CF patients, focusing on genetic factors and mechanisms that may influence viral infection and outcomes. (**A**) ACE2 polymorphisms in CF patients can affect their susceptibility to SARS-CoV-2. Some variants enhance the S protein binding, increasing susceptibility, while others reduce binding and provide protection. (**B**) ACE genotypes impact respiratory disease severity. The ACE (D/D) genotype raises ACE activity, promoting inflammation and severe symptoms, while the ACE (I/I) genotype has the opposite effect, reducing inflammation. CF cells show elevated ACE2 levels, which promote viral binding but also lead to the conversion of proinflammatory Ang-II to anti-inflammatory Ang-1-7, mitigating lung damage. CF patients may have a complex but potentially protective response to SARS-CoV-2 due to specific genetic factors affecting viral interaction and immune response.

Several variants of the ACE2 gene have been identified in previous studies, suggesting that ACE2 polymorphisms may influence host susceptibility to SARS-CoV-2 by altering the ACE2-S protein interaction and/or ACE2 expression levels [63]. Variants such as S19P, I21V, E23K, K26R, T27A, N64K, T92I, Q102P, and H378R are predicted to enhance binding to the S protein, thereby increasing susceptibility to infection [63]. On the other hand, variants including K31R, N33I, H34R, E35K, E37K, D38V, Y50F, N51S, M62V, K68E, F72V, Y83H, G326E, G352V, D355N, Q388L, and D509Y are expected to reduce binding to the S protein [63]. A previous study conducted by Benetti et al. confirmed that some of the ACE2 variants exhibit a direct effect on SARS-CoV-2 spike binding, including p. Leu351Val and p. Pro389His [64]. These variants were found to interfere with the internalization process of the virus in host cells in the Italian population [64]. The genetic variations of ACE2 may contribute to variable respiratory symptoms in individuals infected with SARS-CoV-2, including those with CF [63]. An independent study has determined that in Africa and the Eastern Mediterranean population, ACE2 genotypes were considered protective against COVID-19 (Figure 5A) [64].

Investigations focusing on interactions at the SARS-CoV-2/ACE2 interface, other than ACE2 structure variations caused by certain mutations, can alter the binding of SARS-CoV-2 in different populations. This was achieved by analyzing various ACE2 missense variants that code for ACE2-K26R, ACE2-I468V, ACE2-R219C, ACE2-K341R, ACE2-D206G, and ACE2-G211R and the strength of their interaction with the S protein of SARS-CoV-2 [65]. The analysis was conducted using molecular dynamics and Monte Carlo sampling, which are dominated by electrostatics. In protein–protein interactions, electrostatic interactions are known to be the dominant factor. The data indicate that there is a potential increase in the strength of the binding of the variants, with K26R demonstrating the least favorable binding and G211R demonstrating the most favorable binding [65]. The original data related to ACE2 missense variants were gathered from multiple projects and databases that collect allele frequencies (AF) including the Single Nucleotide Polymorphism Database (dbSNP), the 1000 genomes project phase 3 (1KGP3), the Allele Frequency Aggregator (ALFA project), the Exome Aggregation Consortium (ExAC), the genome Aggregation Database (gnomAD), the Go exome sequencing project (ESP), Trans-Omics for Precision Medicine (TopMed), and a study reporting data from the China Metabolic Analytics Project (ChinaMAP) and other populations [65].

Different computational approaches have been used to predict the differential affinity of a number of ACE2 missense variants for spike proteins. ACE2 is known to be a regulator in the renin–angiotensin–aldosterone system; therefore, ACE2 missense variants or expression quantitative trait loci (eQTL) variants may contribute to pulmonary and systemic injury by promoting vasoconstriction, inflammation, oxidation, and fibrosis, which affect the clinical outcome [66]. The potential association between particular ACE2 gene variants and COVID-19 severity, susceptibility, and clinical outcomes is supported by massive genomic data from the general population. To tightly establish the causal linkage, large-scale genome-wide association studies are needed [66]. A study was performed using in silico molecular docking to analyze the potential effects of ACE2 single-nucleotide polymorphisms (SNPs) resulting in missense variants on the interaction between ACE2 and SARS-CoV-2 spike protein. The results showed that 6 out of the 25 ACE2 missense variants (24%), including I21T, A25T, K26R, E37K, T55A, and E75G, exhibited higher affinity for SARS-CoV-2 spike protein RBD with respect to wild-type ACE2. Moreover, 11 variants (44%), including I21V, E23K, K26E, T27A, E35K, S43R, Y50F, N51D, N58H, K68E, and M82I, showed lower affinity in silico [66].

CF patients with a homozygous deletion in the ACE gene (D/D genotype) tend to experience severe, early-onset respiratory disease and increased inflammation, while patients without this deletion (I/I genotype) generally exhibit better lung function due to reduced ACE activity [63]. ACE and ACE2 are considered opposite players in the balance that determines the risk of developing cardiovascular disease and hypertension [67]. This is related to the fact that ACE catalyzes the conversion of angiotensin-I (Ang-I) to Ang-II, while ACE2 hydrolyzes Ang-II into Ang-1-7 [67]. Ang-II leads to fibrosis, inflammation, and vasoconstriction, while Ang-1-7 leads to increased vasodilation, reduced fibrosis, and inflammation [67]. Data from a CF cohort of 180 patients showed genotype frequencies of 40% D/D, 47% D/I, and 13% I/I [63]. The D/D genotype increases ACE activity, shifting the ACE/ACE2 balance toward higher ACE levels and Ang II production, which intensifies respiratory symptoms and promotes proinflammatory cytokine release [63]. This aligns with the hypothesis that decreased ACE2 activity exacerbates lung inflammation (Figure 5B) [63].

A previous study analyzed publicly available gene microarray data to determine whether ACE2 and TMPRSS2 gene expression is altered in CF patients [63]. This could explain the less severe outcomes in CF patients infected with SARS-CoV-2 compared to the general population, as changes in ACE2 levels influence infection severity [63]. The analysis revealed that ACE2 mRNA is elevated while TMPRSS2 mRNA is reduced in CF airway epithelial cells compared to non-CF cells [63]. While this may seem inconsistent with the earlier mention of ACE2 downregulation in CF patients (Section 5.2), the variation in ACE2 expression can be explained by differences in measurement methods (mRNA vs. protein), post-transcriptional regulation, and model-specific factors such as CFTR mutation type and infection timing. Therefore, increased mRNA levels do not always lead to more functional ACE2 protein, as CFTR defects can impair its processing and surface expression [68]. Although increased ACE2 levels could enhance SARS-CoV-2 binding to epithelial cells, they also promote the conversion of proinflammatory ANG II to anti-inflammatory angiotensin-1-7, potentially reducing lung inflammation and damage [69]. In addition, decreased TMPRSS2 levels may limit SARS-CoV-2 entry into airway epithelial cells since it can cleave ACE2 to enhance viral uptake [63,69]. Taken together, these findings suggest that mutations in the CFTR gene may modulate the abundance of ACE2 and TMPRSS2 proteins, mitigating the severity of SARS-CoV-2-induced lung damage [63].

### 6.2. Host Proteins and SARS-CoV-2 Interactions

Multiple experimental studies have been conducted to predict potential interactions between SARS-CoV-2 RNA and host proteins [70]. Some of these studies have mapped the SARS-CoV-2 RNA–protein interactome across various in vitro-infected cell lines, while others have investigated the functional roles of host proteins involved in these interactions [70]. The findings reveal that the majority of these proteins are involved in regulating viral entry into host cells, protecting host cells from virus-induced cell death, or influencing SARS-CoV-2 pathogenicity [70]. In one study, a multi-omics approach was used to investigate the influence of SARS-CoV-2 on the transcriptome, proteome, ubiquitinome, and phosphoproteome of a lung-derived human cell line [71]. An improved understanding of the mechanisms of SARS-CoV-2 infection was achieved by collecting structural information on SARS-CoV-2 proteins and their interactions with human proteins besides other viral proteins [71]. Additionally, several studies have identified regions within the SARS-CoV-2 genome that may serve as targets for small interfering RNAs (siRNAs) that are considered RNA silencers or interact with host microRNAs (miRNAs) [70].

Omics studies have further characterized COVID-19 phenotypes by integrating virus–host transcriptomics and proteomics in multilayer analysis [70]. Some investigations employed multi-omics approaches to highlight key host components affected by SARS-CoV-2 infection [70]. These studies emphasized the importance of examining the expression of host entry factors, including ACE2 and TMPRSS2, in human tissues [70]. Interestingly, the data indicate that ACE2 expression is extremely low or even absent in tissues commonly targeted by SARS-CoV-2, such as the lungs, bronchus, and nasal mucosa, suggesting a dynamic regulation of entry factors during infection and pointing to the potential involvement of alternative receptors [70]. Two studies specifically investigated the interaction between SARS-CoV-2 and host proteins during viral entry and subsequent replication steps [70]. One study identified host membrane proteins, such as ATP6V1A, AP3B1, STOM, and ZDHHC5, that may facilitate the binding of SARS-CoV-2 structural proteins [70]. Furthermore, several miRNAs were discovered to inhibit proteins involved in viral entry [70]. In this study, a proximity-dependent biotinylation (BioID) approach was applied to identify proximity partners for SARS-CoV-2 proteins in the proteomics “workhorse” 293-cell system [72]. The mapping project provides an abundant resource that can be used by the scientific community for a greater understanding of SARS-CoV-2 pathobiology and identifying virus–host membrane protein interactions that could be COVID-19 therapeutics targets, significantly expanding knowledge regarding the SARS-CoV-2 virus–host interactome [72].

### 6.3. SMN1 and ACE/ACE2 Interactions

A previous study found that tumor necrosis factor (TNF) can suppress ACE expression [73]. However, ACE gene polymorphisms may influence the severity of CF [74]. CXCL8, TNF, and CFTR were identified as key mediators connecting the survival motor neuron 1 (SMN1) with ACE. Using integrated bioinformatic analysis, this study explored the biological interactions between SMN1 and ACE/ACE2 [75]. The findings indicate that ACE2 is essential for SARS-CoV-2 entry into cells and, at the same time, plays a protective role against lung injury [75]. An eQTL analysis revealed that SMN1 deficiency may increase ACE2 levels, potentially providing protection against severe SARS-CoV-2 infection [75]. One study was conducted to highlight a novel regulatory function of SMN in NF-κB signaling. This was achieved by investigating the potential action of SMN on IL-1β-induced NFκB signaling events [76]. The study results showed that SMN1 inhibits the activation of IKK through inhibiting the E3 ligase activity of TRAF6 as well as the kinase activity of IKK [76]. Taking all the findings together, the study proposes that SMN functions as a previously unrecognized inhibitor of the NF-κB inflammatory signaling pathway in the immune cells [76]. Furthermore, individuals with the GG genotype at the single-nucleotide polymorphism rs145558104 of the SMN1 gene exhibited significantly higher SMN1 mRNA expression across SMN1-expressing tissues and lower ACE2 expression in human tissues [75].

These findings highlight the potential functional and signaling interactions between SMN1 (the gene responsible for spinal muscular atrophy) and ACE/ACE2. Given that ACE2 serves as the key receptor mediating SARS-CoV-2 entry into host cells and reduced ACE activity may contribute to COVID-19 severity, understanding this relationship is crucial [75].

## 7. Conclusions

In conclusion, the protective factors and mechanisms that may confer resistance to SARS-CoV-2 infection in CF patients remain incompletely understood and warrant further investigation. Multiple studies suggest that ACE2 polymorphisms in CF patients could influence host susceptibility to SARS-CoV-2. While increased ACE2 expression is associated with enhanced viral binding to epithelial cells, it also facilitates the conversion of ANG II to angiotensin-1-7, which has anti-inflammatory properties. This dual role suggests that elevated ACE2 levels in CF patients may mitigate inflammation and lung damage associated with SARS-CoV-2 infection. Additionally, reduced TMPRSS2 expression in CF patients may limit viral entry into airway epithelial cells. Given these findings, continued research into specific factors and mechanisms contributing to SARS-CoV-2 resistance in CF patients is essential for a comprehensive understanding of this phenomenon. Moreover, studies of COVID-19 vaccine response in CF patients and possible precision medicine studies on ACE2/TMPRSS2 modulation should be conducted in the future.

## Abbreviations

The following abbreviations are used in this manuscript:

SARS-CoV-2	Severe acute respiratory syndrome coronavirus 2
COVID-19	Coronavirus disease 2019
ARDS	Acute respiratory distress syndrome
CF	Cystic fibrosis
CFTR gene	Cystic fibrosis transmembrane conductance regulator gene
ACE	Angiotensin-converting enzyme
TMPRSS2	Transmembrane serine protease 2
DNA	Deoxyribonucleic acid
RNA	Ribonucleic acid
mRNA	Messenger RNA
PFT	Pulmonary function test
CT	Computed tomography
IgM/G	Immunoglobulin M/G
cAMP	Cyclic adenosine monophosphate
TGFB1	Transforming growth factor beta 1
COPD	Chronic obstructive pulmonary disease
NBD1	Nucleotide-binding domain 1
WHO	World Health Organization
RSV	Respiratory syncytial virus
BAL	Bronchoalveolar lavage
IL-1/-6	Interleukin-1/-6
NE	Neutrophil elastase
HRV	Human rhinovirus
HMPV	Human metapneumovirus
FEV1	Forced expiratory volume in 1 s
AE	Asthma exacerbation
RNA-Seq	RNA sequencing
AECs	Airway epithelial cells
IAV	Influenza A virus
HA	Hemagglutinin
NA	Neuraminidase
HPIVs	Human parainfluenza viruses
LRTIs	Lower respiratory tract infections
CD4	Helper T cells
CD8	Cytotoxic T cells
ssRNA	Single-stranded RNA
Orfs	Open reading frames
CDC	Center for Disease Control and Prevention
RdRp	RNA-dependent RNA polymerase
S glycoprotein	Spike glycoprotein
HE	Hemagglutinin-esterase
CoVs	Coronaviruses
MERS-CoV	Middle east respiratory syndrome coronavirus
CME	Clathrin-mediated endocytosis
CNS	Central nervous system
PNS	Peripheral nervous system
BBB	Blood–brain barrier
ATP	Adenosine triphosphate
PaO_2_	Arterial oxygen pressure
Ang-I/-II	Angiotensin I/-II
Ang-1-7	Angiotensin-1-7
siRNAs	Small interfering RNAs
miRNAs	Micro RNAs
BioID	Proximity-dependent biotinylation
TNF	Tumor necrosis factor
SMN1	Survival motor neuron 1
